# Olanzapine-Induced Hyperprolactinemia: Two Case Reports

**DOI:** 10.3389/fphar.2019.00846

**Published:** 2019-07-29

**Authors:** Pedro Cabral Barata, Mário João Santos, João Carlos Melo, Teresa Maia

**Affiliations:** Departamento de Psiquiatria, Hospital Prof. Dr. Fernando da Fonseca, EPE, Amadora, Portugal

**Keywords:** hyperprolactinemia, olanzapine, olanzapine interactions, aripiprazole, fluoxetine

## Abstract

**Background:** Hyperprolactinemia is a common consequence of treatment with antipsychotics. It is usually defined by a sustained prolactin level above the laboratory upper level of normal in conditions other than that where physiologic hyperprolactinemia is expected. Normal prolactin levels vary significantly among different laboratories and studies. Several studies indicate that olanzapine does not significantly affect serum prolactin levels in the long term, although this statement has been challenged.

**Aims:** Our aim is to report two olanzapine-induced hyperprolactinemia cases observed in psychiatric consultations.

**Methods:** Medical records of the patients who developed this clinical situation observed in psychiatric consultations in the Psychiatry Department of the Prof. Dr. Fernando Fonseca Hospital during the year of 2017 were analyzed, complemented with a non-systematic review of the literature.

**Results:** The case reports consider two women who developed prolactin-related symptoms after the initiation of olanzapine. No baseline prolactinemia was obtained, and prolactin serum levels were only evaluated after prolactin-related symptoms developed: at the time of its measurement, both patients had been taking olanzapine for more than 24 weeks. Hyperprolactinemia was found to be present in Case 2, whereas Case 1 (a 49-year-old woman) had “normal” serum prolactin levels for premenopausal and prolactin levels slightly above the maximum levels for postmenopausal women. Both patients underwent similar pharmacological adjustments, which comprised switches from olanzapine to aripiprazole. After all pharmacological changes, prolactin serum levels decreased to normal range values and prolactin-related symptoms disappeared.

**Discussion/Conclusions:** Laboratorial and literature prolactinemia values variability and discrepancies may make the management of borderline hyperprolactinemia clinical situations difficult. Baseline prolactin levels should have been obtained, as they help in the management of patients who develop neuroleptic-induced hyperprolactinemia. Prolactin-related symptoms can occur with borderline or normal standardized prolactinemia values. Olanzapine-induced hyperprolactinemia is a rare but possible event. Aripiprazole was used as a suitable alternative for olanzapine-induced hyperprolactinemia.

## Introduction

Hyperprolactinemia is a common (Yasui-Furukori et al., 2010; Montejo et al., 2016; Montejo et al., 2017) and underappreciated consequence of the treatment with antipsychotic drugs (Montejo et al., 2016; Montejo et al., 2017). It is usually defined by a sustained prolactin level above the laboratory upper level of normal in conditions other than those where physiologic hyperprolactinemia is expected (e.g., pregnancy and lactation) (Peuskens et al., 2014; Montejo et al., 2017). Prolactin is a polypeptide hormone that is mainly synthesized and secreted from lactotroph cells of the anterior lobe of the pituitary gland. Normal prolactin levels range at 10–20 ng/ml for men and 10–25 ng/ml for women, although there is significant variability among different laboratories and studies (Peuskens et al., 2014). Furthermore, there are important interindividual differences (e.g., gender, age, stress, nutrition, and exercise) (Peuskens et al., 2014; Yang et al., 2018) and pronounced circadian variations (Peuskens et al., 2014). Hyperprolactinemia can be divided according to its severity: mild (50 ng/ml), moderate (51–75 ng/ml), and severe (>100 ng/ml) (Montejo et al., 2016).

In women, hyperprolactinemia can cause amenorrhea, galactorrhea, cessation of normal cyclic ovarian function, and hirsutism. In men, it can cause gynecomastia, impotence, loss of libido, and hypospermatogenesis. Long-term hypogonadism due to hyperprolactinemia has also been associated with low bone density, osteoporosis, hip fracture (Yasui-Furukori et al., 2010; Pérez-Iglesias et al., 2012; Montejo et al., 2016), prolactinoma (Montejo et al., 2017), and increased cardiovascular risk (Montejo et al., 2016; Montejo et al., 2017). Women are considered to be more susceptible to be affected by antipsychotic-induced hyperprolactinemia; in spite of that, there is little literature reporting the trajectory of olanzapine-induced hyperprolactinemia in females (Yang et al., 2018). Preclinical animal models indicate that prolonged high levels of prolactin may predispose to breast cancer (Pérez-Iglesias et al., 2012); what is more, recent clinical studies have strengthened such association. The symptoms that accompany prolactin elevation not only are unpleasant but also contribute to increased stigma (e.g., gynecomastia in males), jeopardizing their health and their treatment adherence (Montejo et al., 2016).

Antipsychotic drugs have differences in their propensity to cause hyperprolactinemia. Several mechanisms have been proposed to explain these variances (Pérez-Iglesias et al., 2012; Montejo et al., 2016; Montejo et al., 2017): D2 receptor-binding affinity (the stronger the dopamine blockade, the higher the prolactin elevation) (Pérez-Iglesias et al., 2012; Peuskens et al., 2014; Montejo et al., 2016; Montejo et al., 2017), differential penetrability across the blood–brain barrier (Pérez-Iglesias et al., 2012; Montejo et al., 2016; Montejo et al., 2017), and central mechanisms modulated by monoamines other than dopamine (Pérez-Iglesias et al., 2012).

Although first-generation antipsychotics are associated with pronounced elevations of prolactin levels (Pérez-Iglesias et al., 2012; Montejo et al., 2016; Montejo et al., 2017), the highest rates of hyperprolactinemia have been reported with sulpiride (Peuskens et al., 2014), amisulpride, risperidone (Peuskens et al., 2014; Montejo et al., 2016; Montejo et al., 2017), and paliperidone (Montejo et al., 2016; Montejo et al., 2017). These rates can be as high as 80–90% and are consistently greater than those found with other neuroleptics (Peuskens et al., 2014). Other second-generation agents are less likely to generate sustained hyperprolactinemia (Yasui-Furukori et al., 2010; Montejo et al., 2016; Montejo et al., 2017). While some authors argue that prolactin response to antipsychotic treatments weakens over time (Yasui-Furukori et al., 2010), others report that serum prolactin levels tend to remain elevated during the length of neuroleptic treatment (Montejo et al., 2017). A Multidisciplinary Consensus Group of Experts has proposed a routine prolactin level measurement to all antipsychotic-treated patients, at baseline and at 3-month time (Montejo et al., 2016). Olanzapine, an antipsychotic with an intermediary D2 receptor binding affinity, induces a moderate elevation of prolactin levels (Peuskens et al., 2014). This elevation is regarded as transient and mild compared with that induced by other antipsychotics, such as risperidone (Yasui-Furukori et al., 2010; Montejo et al., 2016), with studies pointing that olanzapine does not significantly affect serum prolactin levels in the long term (Pérez-Iglesias et al., 2012; Montejo et al., 2016). There have been inconsistent results regarding the relationship between serum prolactin levels and doses/plasma concentrations of olanzapine (Peuskens et al., 2014; Takeuchi et al., 2014), although most studies have demonstrated a dose-dependent effect of oral olanzapine on the plasma prolactin level of patients with schizophrenia, with higher doses linked to higher prolactin levels (Peuskens et al., 2014; Takeuchi et al., 2014; Yang et al., 2018). All things considered, prolactin elevation induced by olanzapine remains an unclear matter (Yang et al., 2018). Some D2 receptor and 5-HT2A receptor gene polymorphisms have been involved in prolactin levels after administration of olanzapine (Peuskens et al., 2014); DRD2 and ANKK1 polymorphisms have been associated with prolactin increase in olanzapine-treated women (Houston et al., 2011).

## Methods

We present two case reports of patients observed in psychiatric consultations in the Psychiatry Department of the Prof. Dr. Fernando Fonseca Hospital during the year of 2017. The case description is done through clinical record analysis. Informed consent was obtained from the participants for the publication of this case report.

A review of the literature considering psychopharmacy-induced hyperprolactinemia and its relationship with olanzapine was also performed: PubMed/MEDLINE database search of scientific articles [MeSH (Medical Subject Headings) terms “hyperprolactinemia,” “olanzapine,” “prolactin,” “olanzapine interactions,” and “fluoxetine”; written in English and Spanish; from the year 1999 to 2018] and the use of psychopharmacology textbooks.

## Case Presentation

Two cases of olanzapine-induced hyperprolactinemia are presented, whose sociodemographic and clinical characteristics can be found in **Tables 1** and **2**.

**Table 1:** Patients’ sociodemographic and basic clinical data.

	Case 1	Case 2
**Age (in years)**	49	29
**Gender**	Female	Female
**Psychiatric diagnosis (ICD-10)**	Bipolar affective disorder (F31)	Delusional disorder (F22.0)
**Somatic comorbidities**	None	None
**Tobacco smoking**	No	No

**Table 2:** Prolactin-related clinical data [laboratorial prolactinemia dose ranges (ng/ml): (6.0–29.9) for pre-menopause and (1.8–20.3) for post-menopause].

	Case 1	Case 2
**Baseline prolactin-related symptoms**	Galactorrhea and breast pain	Galactorrhea, breast tension, and amenorrhea
**Baseline medication**	Fluoxetine 20 mg/day Olanzapine 5 mg/day	Olanzapine 20 mg/day Aripiprazole 2.5 mg/day
**Pharmacological changes**	Switch olanzapine–aripiprazole through partial overlap (switch duration: 1 month)	Switch olanzapine 20 mg/day to aripiprazole 20 mg/day through partial overlap (switch duration: 2 months)
**Psychiatric disorder relapse**	No	Yes
**Second pharmacological modification due to relapse**	N/A	Initiation of aripiprazole injectable formulation 400 mg every 4 weeks. Gradual stoppage of aripiprazole oral 20 mg/day
**Medication after all pharmacological adjustments**	Fluoxetine 20 mg/day Aripiprazole 20 mg/day	Aripiprazole injectable formulation 400 mg every 4 weeks
**Prolactinemia before pharmacological adjustments (ng/ml)**	20.73	50
**Prolactinemia after pharmacological all adjustments (ng/ml)**	7.27	8.51
**Prolactin-related symptoms after all pharmacological adjustments**	Asymptomatic	Asymptomatic
**Psychiatric disorder after all pharmacological adjustments**	Stable	Stable

Both patients developed prolactin-related symptoms after the initiation of olanzapine. These patients complained about the symptoms at their psychiatric consultations, referring that they were significantly disturbing their quality of life. Prolactinemia levels were only obtained after these complaints, as there were no asymptomatic prolactin serum levels. At the time when prolactinemia levels were obtained, both patients had been taking olanzapine for more than 24 weeks.

Patient 1 required one pharmacological adjustment (from oral olanzapine 5 mg/day to oral aripiprazole 20 mg/day), allowing prolactin-related symptoms to disappear and prolactin serum levels to diminish while maintaining psychiatric stability.

Patient 2, on the other hand, showed a symptomatic recrudescence from her psychiatric condition after the psychopharmacological modification (oral olanzapine 20 mg/day to oral aripiprazole 20 mg/day), with the resurgence of autoreferential delusion and olfactory hallucinations. Such clinical decompensation demanded further pharmacological changes, with the initiation of an injectable formulation of aripiprazole (400 mg, every 4 weeks) and gradual stoppage of oral aripiprazole (20 mg per day). This second adjustment allowed for the remission of psychiatric symptoms and simultaneous achievement of psychiatric stability. After all pharmacological changes, prolactin serum levels decreased to normal range values and prolactin-related symptoms vanished.

## Discussion

The reference prolactinemia levels available in scientific literature did not match with the laboratory values from the hospital where these patients were followed up—a discrepancy that may prove problematic when managing borderline hyperprolactinemia clinical situations. Nevertheless, it is important to bear in mind that these “normal” range levels have been found to be significantly variable among different laboratories and studies (Peuskens et al., 2014).

In Case 1, there was a prolactin increase with only 5 mg of olanzapine per day. Being aware that the increases in prolactin serum levels induced by olanzapine are uncommon and dose related, one might think that the reason behind it would be related with its interaction with fluoxetine. However, it is known that the changes in olanzapine pharmacokinetics from its coadministration with fluoxetine are clinically insignificant (Callaghan et al., 1999). What is more, even though there were prolactin-related symptoms (which disappeared after prolactin serum levels fell), the prolactinemia levels in this patient were considered “normal” for premenopausal and slightly above the maximum levels for postmenopausal women. Taking into account her age (49 years), her prolactin levels, and her prolactin-related symptom evolution, a possible explanation is that this patient was perimenopausal. Otherwise, we may just be sitting in front of a patient with lower normal-range values for prolactinemia when compared with those of general population; or it may just be the reflection of the laboratorial value variability.

The clinical aggravation in Case 2 was possibly related with the switch from 20 mg of olanzapine to 20 mg of aripiprazole. According to the literature, 20 mg of olanzapine is equivalent to 30 mg of aripiprazole (Taylor et al., 2018). Therefore, this psychopharmacological change leads to a lower antipsychotic dosage, a probable cause of symptom worsening. After a further dosage adjustment (oral aripiprazole 20 mg/day to injectable aripiprazole 400 mg every 4 weeks), the patient became psychiatrically asymptomatic.

Another possible cause for Case 2 relapse may have been due to a switch being done between a dopamine receptor antagonist (olanzapine) and a dopamine receptor partial agonist (aripiprazole), together with the fact that aripiprazole strongly binds to dopamine receptors, displacing almost every other antipsychotic and stimulating receptors from minimal dopamine activity to about 30%, which can be acutely distressing and aversive (Bazire, 2014).

Olanzapine-induced prolactin serum level elevation has been described as transient, equaling placebo at week 6 (Callaghan et al., 1999; Montejo et al., 2016). However, both patients were medicated with olanzapine for more than 24 weeks, a fact that reinforces their clinical atypicality and, consequently, their scientific importance.

Contrary to what is suggested in the literature, there were no baseline plasma prolactin levels, as they were only obtained after patients complained of prolactin-related symptoms. If these levels had been collected, an earlier pharmacological intervention might have been possible, perhaps lessening the appearance, duration, or disturbance caused by prolactin-related symptoms.

The switch to aripiprazole, as advised in the literature (Montejo et al., 2016; Montejo et al., 2017), was a successful approach in both patients, not only diminishing serum prolactin values but also solving prolactin-related symptoms and maintaining clinical stabilization.

## Conclusion

Literature prolactinemia values do not always correspond with laboratory values from clinical practice, a discrepancy that may make the management of borderline hyperprolactinemia clinical situations difficult. Baseline prolactin level collection may help in the management of patients who develop neuroleptic-induced hyperprolactinemia. Prolactin-related symptoms can occur with borderline or normal standardized prolactinemia values. Olanzapine-induced hyperprolactinemia is a rare event, but it is not nonexistent. Aripiprazole was used as a suitable alternative for olanzapine-induced hyperprolactinemia.

## Data Availability

All datasets generated for this study are included in the manuscript and/or supplementary files.

## Ethics Statement

This study was approved by the ethical committee of the investigators Hospital. Written informed consent was obtained from the individual(s) for the publication of any potentially identifiable images or data included in this article.

## Author Contributions

PB contributed to the conception and design of the study. PB and MS wrote the first draft of the manuscript. All authors contributed to manuscript revision and edition and read and approved the submitted version.

## Funding

The authors declare that this study received funding from Lundbeck Pharmaceutical Company, as it subsidized the author publication fee. The funder had no role in study design, data collection and analysis, decision to publish, or preparation of the manuscript.

## Conflict of Interest Statement

The authors declare that the research was conducted in the absence of any commercial or financial relationships that could be construed as a potential conflict of interest.

## References

[B1] Bazire S. (2014). “Switching or discontinuing psychotropics,” in Psychotropic drug directory. Ed. Bazire S (Dorsington: Lloyd-Reinhold Communications LLP).

[B2] Callaghan J. T. Bergstrom R. F. Ptak L. R. Beasley C. M. (1999). Olanzapine: pharmacokinetic and pharmacodynamic profile. Clin. Pharmacokinet. 37 (3), 177–193. 10.2165/00003088-199937030-00001 10511917

[B3] Houston J. Dharia S. Bishop J. R. Ellingrod V. L. Fijal B. Jacobson J. G. (2011). Association of DRD2 and ANKK1 polymorphisms with prolactin increase in olanzapine-treated women. Psychiatry Res. 187 (1–2), 74–79. 10.1016/j.psychres.2010.10.020 21095016

[B4] Montejo ÁL Arango C. Bernardo M. Carrasco J. L. Crespo-Facorro B. Cruz J. J. (2016). Spanish consensus on the risks and detection of antipsychotic drug-related hyperprolactinaemia. Rev. Psiquiatr. Salud Ment. 9 (3), 158–173. 10.1016/j.rpsm.2015.11.003 26927534

[B5] Montejo ÁL Arango C. Bernardo M. Carrasco J. L. Crespo-Facorro B. Cruz J. J. (2017). Multidisciplinary consensus on the therapeutic recommendations for iatrogenic hyperprolactinemia secondary to antipsychotics. Front. Neuroendocrinol. 45, 25–34. 10.1016/j.yfrne.2017.02.003 28235557

[B6] Pérez-Iglesias R. Mata I. Martínez-García O. Garcia-Unzueta M. T. Amado J. A. Valdizán E. M. (2012). Long-term effect of haloperidol, olanzapine, and risperidone on plasma prolactin levels in patients with first-episode psychosis. J. Clin. Psychopharmacol. 32 (6), 804–808. 10.1097/JCP.0b013e318272688b 23131886

[B7] Peuskens J. Pani L. Detraux J. De Hert M. (2014). The effects of novel and newly approved antipsychotics on serum prolactin levels: a comprehensive review. CNS Drugs 28 (5), 421–453. 10.1007/s40263-014-0157-3 24677189 PMC4022988

[B8] Takeuchi H. Suzuki T. Remington G. Watanabe K. Mimura M. Uchida H (2014). Lack of effect of risperidone or olanzapine dose reduction on metabolic parameters, prolactin, and corrected QT interval in stable patients with schizophrenia. J. Clin. Psychopharmacol. 34 (4), 517–520. 10.1097/JCP.0000000000000142 24911439

[B9] Taylor DM Barnes TRE Young AH, editors. (2018). “Akathisia,” in The Maudsley Prescribing guidelines in psychiatry, 13th ed (Chichester: John Wiley & Sons Ltd), 94–97.

[B10] Yang F. Chen L. Fang X. Zheng K. Zhu C. Xu C. (2018). Influence of olanzapine on serum prolactin levels and BMI in female patients with schizophrenia. Neuropsychiatr. Dis. Treat. 14, 3373–3379. 10.2147/NDT.S180303 30587989 PMC6298388

[B11] Yasui-Furukori N. Saito M. Nakagami T. Sugawara N. Sato Y. Tsuchimine S. (2010). Gender-specific prolactin response to antipsychotic treatments with risperidone and olanzapine and its relationship to drug concentrations in patients with acutely exacerbated schizophrenia. Prog. Neuropsychopharmacol. Biol. Psychiatry 34 (3), 537–540. 10.1016/j.pnpbp.2010.02.014 20170699

